# Risk factors for total joint arthroplasty infection in patients receiving tumor necrosis factor α-blockers: a case-control study

**DOI:** 10.1186/ar3087

**Published:** 2010-07-16

**Authors:** Mélanie Gilson, Laure Gossec, Xavier Mariette, Dalenda Gherissi, Marie-Hélène Guyot, Jean-Marie Berthelot, Daniel Wendling, Christian Michelet, Pierre Dellamonica, Florence Tubach, Maxime Dougados, Dominique Salmon

**Affiliations:** 1 Rheumatology B Department, Cochin Hospital, AP-HP, 27 rue du faubourg Saint-Jacques, Paris 75014, France; UPRES-EA 4058, Medicine Faculty, Paris Descartes University, 12 rue de l'Ecole de Médecine, Paris 75006, France; 2 Department of Rheumatology, Bicêtre Hospital, AP-HP, 78 rue du Général Leclerc, Le Kremlin-Bicêtre 94270, France; INSERM U802, Paris-Sud University, 63 rue Gabriel Péri, Le Kremlin-Bicêtre 94270, France; 3 Infectious Diseases - Internal Medicine Department, Cochin Hospital, AP-HP, 27 rue de faubourg Saint-Jacques, Paris 75014, France; 4 Department of Rheumatology, Provo Hospital, 25 rue de Barbieux, Roubaix 59100, France; 5 Department of Rheumatology, Hotel Dieu Hospital, 1 place Alexis-Ricordeau, Nantes 44000, France; 6 Department of Rheumatology, Minjoz Hospital, 3 boulevard Alexandre Fleming, Besançon 25000, France; 7 Department of Infectious Diseases, University Hospital, 2 rue de l'Hôtel-Dieu, Rennes 35000, France; 8 Department of Infectious Diseases, University Hospital, 4 avenue Reine Victoria, Nice 06000, France; 9 Department of Clinical Epidemiology and Biostatistics, Bichat Hospital, AP-HP, 46 rue Henri Huchard, Paris 75018, France; INSERM U738, Medicine Faculty, Paris 7 Denis Diderot University, 16 rue Henri Huchard, Paris 75018, France

## Abstract

**Introduction:**

The objective of this study was to assess natural microbial agents, history and risk factors for total joint arthroplasty (TJA) infections in patients receiving tumor necrosis factor (TNF)α-blockers, through the French RATIO registry and a case-control study.

**Methods:**

Cases were TJA infections during TNFα-blocker treatments. Each case was compared to two controls (with TJA and TNFα-blocker therapy, but without TJA infection) matched on age (±15 years), TJA localization, type of rheumatic disorder and disease duration (±15 years). Statistical analyses included univariate and multivariate analyses with conditional logistic regression.

**Results:**

In the 20 cases (18 rheumatoid arthritis), TJA infection concerned principally the knee (*n *= 12, 60%) and the hip (*n *= 5, 25%). *Staphylococcus *was the more frequent microorganism involved (*n *= 15, 75%). Four patients (20%) were hospitalized in an intensive care unit and two died from infection. Eight cases (40%) versus 5 controls (13%) had undergone primary TJA or TJA revision for the joint subsequently infected during the last year (*P *= 0.03). Of these procedures, 5 cases versus 1 control were performed without withdrawing TNFα-blockers (*P *= 0.08). In multivariate analysis, predictors of infection were primary TJA or TJA revision for the joint subsequently infected within the last year (odds ratio, OR = 88.3; 95%CI 1.1-7,071.6; *P *= 0.04) and increased daily steroid intake (OR = 5.0 per 5 mg/d increase; 1.1-21.6; *P *= 0.03). Case-control comparisons showed similar distribution between TNFα-blockers (*P *= 0.70).

**Conclusions:**

In patients receiving TNFα-blockers, TJA infection is rare but potentially severe. Important risk factors are primary TJA or TJA revision within the last year, particularly when TNFα-blockers are not interrupted before surgery, and the daily steroid intake.

## Introduction

The efficacy of TNFα blocker is now well established in patients with rheumatoid arthritis (RA) [1], ankylosing spondylitis (AS) [2] and psoriatic arthritis (PsA) [3]. Consequently, the prescription of these drugs becomes more and more frequent. Their use in patients with rheumatic disorders has led to less joint destruction and patients' functional prognosis has been greatly improved [4-6]. The requirement for total joint arthroplasty (TJA) tended to decrease in rheumatic patients before the use of TNFα blockers in rheumatology, thanks to strategies of earlier and more intensive management of recent rheumatic disorders [7,8]. The use of biologic treatments, and in particular of TNFα blockers, in rheumatic disorders will probably increase this downward trend. However, the need for TJA remains frequent, particularly due to joint destructions occurring before the introduction of TNFα blockers. Moreover, many patients already have one or more TJA at the time of TNFα blockers introduction.

The increased risk of tuberculosis and other opportunistic infections in patients receiving TNFα blockers is now well known [9,10]. An increased risk of serious bacterial infections in RA patients receiving TNFα blockers has also been established through two meta-analyses of randomized controlled trials [11,12] and retrospective cohort studies [13,14], although other studies gave contradictory results [15,16].

One of the most severe complications of TJA is surgical site infection, leading to long and expensive hospitalizations, complicated additional surgical procedures, increased mortality rates and severe functional disability. Despite systematic preventive measures, the risk of TJA infection persists and has been estimated at 1% for total hip arthroplasty and 2% for total knee arthroplasty [17-19]. Moreover, a two- to four-fold increased risk has been reported in RA [20,21], although not found in other studies [22]. The role of treatments and particularly TNFα blockers in this increased risk remains unclear [23]. Some studies concluded a similar risk of postoperative infection after orthopedic surgery whether the patients were exposed or not to TNFα blockers [24-26], whereas other studies highlighted a higher risk with TNFα blockers [27,28] reaching a two-fold increase [28]. However, these data remain controversial.

Other identified risk factors of TJA infections are systemic malignancy [29], previous prosthetic joint infection of the index joint and of any joint [21], arthroplasty revision [21,29], increased operative time [21] and postoperative surgical site infection not involving the arthroplasty [29]. Nothing is known about the relevance of these risk factors in patients exposed to TNFα blockers.

The objectives of the present study were to evaluate the microbial agents, natural history and risk factors of TJA infections in patients receiving TNFα blockers, through a case-control study.

## Materials and methods

### Study design

This was a case-control study including cases recruited from a national registry (Research Axed on Tolerance of bIOtherapies (RATIO) registry) and controls retrospectively recruited from a tertiary care centre. The RATIO registry was authorized by the ethical committee of AP-HP, GHU Nord (Institutional Review Board of Paris North Hospitals, Paris 7 University, AP-HP; authorization number 162-08) [30]. Data concerning controls and issued from their usual planned visits were collected retrospectively and analyzed anonymously; no ethical approval is necessary for this type of analysis in France (Huriet-Sérusclat law: law n°88-1138; 20 December, 1988; published in the Journal Officiel on 22 December, 1988). Natural history of TJA infections in patients exposed to TNFα blockers was described. To assess risk factors of TJA infections, each case was compared with two matched controls.

### Cases

Cases had a rheumatic disorder (RA, AS, or PsA) treated with TNFα blockers. They presented with TJA infection while exposed to TNFα blockers or less than one year after their withdrawal. Only TJA of large joints were considered (hip, knee, ankle, shoulder, or elbow) whatever their indication (the rheumatic disorder itself, osteoarthritis, or other cause). Each case was validated by an expert committee; a positive culture report was not mandatory to define TJA infection for the purposes of this study if clinical, biologic, morphologic, or histologic features highly supported the diagnosis. Cases were principally recruited through the national RATIO registry. In this registry opportunistic infections, severe bacterial infections and lymphomas complicating TNFα-blocker therapies were prospectively collected in France between 1 February, 2004 and 31 January, 2006 [31]. Cases were self-reported by clinicians in rheumatology departments, departments of infectious diseases, orthopedic departments, and ICUs all over the country in specific clinical research forms. Access to clinical files was possible. To collect more cases, all infectious diseases physicians from the French Society of Infectious Diseases and 1,800 rheumatologists from French hospital centers prescribing TNFα blockers in rheumatic diseases, and registered on "Club Rheumatism and Inflammation", a section of the French Society of Rheumatology were contacted through their respective web sites [32,33], and received repeated e-mails to obtain the files of patients with TNFα-blocker-induced TJA infection between 1 February, 2006 and 30 April, 2008. Clinical data (e.g., disease activity) were collected for the period of the diagnosis of TJA infection. Follow up was continued at least until the end of the antibiotic treatment. A 12-month follow up after TJA infection was deemed necessary to confirm termination of the infection; otherwise the infection outcome was categorized as unknown.

### Controls

For each case, two controls were recruited, with TJA and TNFα-blocker treatment but without TJA infection. Prespecified matching criteria were age ±15 years, same underlying rheumatic disorder, rheumatic disorder duration ±15 years, and same TJA localization. All was performed during the selection of controls to find in our cohort of patients for each case the two controls best fulfilling the matching criteria. In case of peripheral PsA, RA controls were accepted. At the beginning of the study, no data published in the literature suggested a different risk of infection, and particularly of prosthetic infection, between females and males. That is the reason why we decided that controls would not be required to be of the same gender as the cases. All controls were retrospectively recruited in the Rheumatology B Department in Cochin Hospital, a tertiary care center, through a computerized search of the data files of outpatients and inpatients between 2002 and 2008. For each control, time of clinical data collection was chosen for best matching of age and disease duration. The impossibility of finding two matched controls was an exclusion criterion of cases.

### Data collection

Data abstracted from the files were noted on a standardized chart review tool. In RA, rheumatic disease activity assessed by the Disease Activity Score 28 [34] was classified as remission (<2.6), low (2.6 to 3.2), moderate (3.2 to 5.1) and high (>5.1) activity. Considering the definition of nosocomial TJA infections (i.e. occurring within the 12 months after TJA setup) [35] and a median time between surgical procedure (primary TJA or TJA revision) and TJA infection of 0.3 years (interquartile range (IQR): 0.1 to 0.8) [21], primary TJA or TJA revision within the past year on the affected or matched joint was reported, taking into account if this surgery had been performed before or after the introduction of TNFα blockers. In the second situation, the patients were considered no longer exposed to TNFα blockers at the time of surgery if TNFα blockers had been withdrawn at least five half-lives before surgery (50 days for infliximab, 70 days for adalimumab, 15 days for etanercept).

### Statistical analysis

Statistical analyses were performed using SAS version 9.1 (SAS France, Domaine de Grégy, Grégy-sur-Yerres, 77257 Brie Comte Robert cedex, France). They included univariate and multivariate analyses with conditional logistic regression to take into account the matching (PHREG procedure). All variables with a *P *value less than 0.20 in univariate analysis were entered in the multivariate regression. Results achieving a *P *value less than 0.05 were considered as statistically significant.

## Results

### Cases

Twenty-two cases of TJA infection in patients treated with TNFα blockers were collected: 13 from the RATIO registry and 9 through the websites of the French Societies of Infectious Diseases and of the Club Rheumatism and Inflammation. Two cases with elbow arthroplasty infection were excluded due to lack of matched controls. Consequently 20 cases were included in the present case-control study (Table 1). Nineteen were female (95%); mean age was 57.3 ± 12.4 years. Eighteen had RA (90%). The other two cases detailed below suffered from other rheumatic diseases. A 43-year-old woman, with AS of 22 years' duration and treated with infliximab, presented an infection due to methicillin-resistant *Staphylococcus aureus *(MRSA) on hip arthroplasty, and a 40-year-old woman, with PsA of 5 years' duration and treated with infliximab, presented an infection due to methicillin-susceptible *Staphylococcus aureus *(MSSA) on knee arthroplasty. Mean duration of rheumatic disorder was 20.4 ± 9.4 years. Seven patients received infliximab (3 mg/kg in four, 5 mg/kg in two, unknown dose in one), five received etanercept (25 mg twice a week in four, 50 mg once a week in one) and eight patients received 40 mg adalimumab every other week. TJA infections concerned the knee (*n *= 12, 60%), the hip (*n *= 5, 25%), the shoulder (*n *= 2), and the ankle (*n *= 1).

**Table 1:** Comparison of cases † and controls* regarding matching criteria (univariate analysis with conditional logistic regression)

	Cases † (*n *= 20)	Controls* (*n *= 40)	P
Age (years) **	57.3 ± 12.4	57.5 ± 10.9	0.89
RA/PsA/AS, n	18/1/1	38/0/2	0.99
Rheumatic disorder duration (years) **	20.4 ± 9.4	20.3 ± 8.5	0.97
TJA infection localization for cases, and matched TJA localization for controls, n (%)			
- Hip	5 (25)	10 (25)	1
- Knee	12 (60)	24 (60)	1
- Ankle	1 (5)	2 (5)	1
- Shoulder	2 (10)	4 (10)	1

† at the time of diagnosis of TJA infection; * time chosen for a best matching; ** mean ± standard deviation.

AS, ankylosing spondylitis; PsA, psoriatic arthritis; RA, rheumatoid arthritis; TJA, total joint arthroplasty.

### Microbial agents

Nineteen cases (95%) had at least one positive microbiological sample, i.e. hemocultures (*n *= 12, 60%), joint fluid (*n *= 10, 50%), non surgical synovial biopsy (*n *= 7, 35%), surgical biopsy (*n *= 7, 35%) and drain (*n *= 2). Two files mentioned histologic findings supporting the diagnosis of infection. In one case, no microbial agent was identified, but the diagnosis of TJA infection was considered as assured considering the association of an acute access of fever and shivering, suppurating joint fluid, C-reactive protein (CRP) level at 438 mg/L, leukocytes level at 17.7 G/L, positive leukoscan and a rapid improvement with ciprofloxacin and cloxacillin. *Staphylococcus *was the most frequent microorganism involved (*n *= 15, 75%), followed by *Streptococci *(*n *= 4: *Streptococcus oralis*, group A *Streptococcus hemolyticus*, group B *Streptococcus*, and **Streptococcus salivarius**), *Escherichia coli *(*n *= 1) and *Enterococcus *(*n *= 1). *S. aureus *was identified in 13 cases (65%) and was most often susceptible to methicillin (*n *= 11, 85%). In the 12 knee arthroplasty infections, involved microbial agents were MSSA in 7 cases, MSSA and *Streptococcus *in 1 case, MRSA and *Enterococcus *in 1 case, coagulase-negative *Staphylococcus *in 1 case, *Streptococcus *in 1 case and none in 1 case. In the 5 hip arthroplasty infections, they were *Streptococcus *in 2 cases, MSSA in 1 case, MRSA in 1 case, and coagulase-negative *Staphylococcus *in 1 case. No opportunistic infection was observed.

### Natural history of TJA infection in patients exposed to TNFα-blockers

All patients were hospitalized, 4 of them (20%) in an ICU. Median delay from the last TNFα blocker administration was 20 ± 68 days (30 days for infliximab, 14 days for etanercept and 10 days for adalimumab). Symptoms appeared suddenly in 7 cases, progressively in 8; the onset mode was unknown in the 5 other cases. Reported symptoms were joint pain (*n *= 16, 80%), swollen joint (*n *= 10, 50%), fever (*n *= 10, 50%), shivering (*n *= 7, 35%), septic shock or severe sepsis (*n *= 5), fistulization (*n *= 2) and iterative TJA dislocation (*n *= 1). Nine other infected sites were identified in 7 cases: urinary infection (*n *= 2), metacarpo-phalangeal arthritis (*n *= 1), psoas abscess in 1 total hip arthroplasty infection, skin infection of homolateral lower limb in 2 total knee arthroplasty infections, abcess of the thigh in 2 total knee arthoplasty infections, and jugal abscess (*n *= 1). Median CRP level was 272 mg/L (range 15 to 502). Median polynuclear neutrophils level was 9.2 G/L (range 4.5 to 14.0). Median lymphocytes level was 0.7 G/L (range 0.2 to 2.0).

Eighteen patients underwent surgical treatment: joint lavage (*n *= 11, 55%), prosthesis extraction without reimplantation at the time of the last news (*n *= 6), and two-stage arthroplasty exchange (*n *= 1). One patient received only medical treatment and the type of surgical treatment was unknown in the last patient. All cases received antibiotic treatment: bi- or multi-antibiotic treatment (*n *= 18, 90%), mono-antibiotic treatment (*n *= 1), not reported in one. In the 18 alive patients, median antibiotic treatment duration in treated patients was 90 days (IQR: 45 to 146), median bi-antibiotic treatment duration was 45 days (IQR: 42 to 112), and median intravenous antibiotic treatment duration was 30 days (IQR: 17 to 45).

Over a median follow-up duration of 14 months (IQR: 5 to 19), infection outcome was death in 2 cases, both occurring during the first month (11^th ^and 22^nd ^day) and related to infection, recovery in 11 cases (55%), infection relapse in 1 case, and unknown in 6 patients. In the 18 alive patients, 67% presented a rheumatic flare (10 of 15 available data) and 7 moderate to severe functional disability of the infected joint (39%). Only one case did not experience any complication regarding the infection outcome, rheumatic disorder outcome and functional prognosis on the infected joint.

TNFα blockers were always withdrawn at the time of TJA infection. In 3 patients, the same TNFα blocker was reintroduced after the recovery from infection 3, 4.5 and 14 months, respectively, after TJA infection diagnosis. Infection relapse occurred in one case where the TNFα blocker was reintroduced at 3 months. TNFα blockers were not reintroduced in 14 cases, because the risk of infection was considered too high (*n *= 12), rheumatic disease remission (*n *= 1) or patient's refusal (*n *= 1). The decision of reintroducing or not TNFα blockers was unknown in the last case.

### Risk factors of TJA infection in univariate analysis

Characteristics of the 20 cases (at the time of diagnosis of TJA infection) and 40 controls (time chosen for a best matching) are compared in Tables 1 and 2. As expected, no difference was observed regarding matching criteria (Table 1). The rheumatic disorder activity and main comorbidities (including diabetes mellitus) were similar in both groups. History of TJA infection before the introduction of TNFα blockers was reported in three cases, two involving the same joint, versus no control (*P *= 0.08). Primary TJA or TJA revision for the joint subsequently infected was performed during the preceding year in eight (40%) cases (of which four primary TJA and three TJA revisions) versus five (13%) controls (of which three primary TJA and two TJA revisions; *P *= 0.03). If the majority of procedures (6/8 in cases, and 5/5 in controls) were performed after the introduction of TNFα blockers, the drug was withdrawn at least five half-lives before surgery in only one of six cases, versus four of five controls (*P *= 0.08). The fact that TNFα blockers were withdrawn or not before this surgery was not entered into the multivariate model because this parameter only concerned the six cases and five controls who had undergone primary TJA or TJA revision after the introduction of TNFα blockers, and not the 20 cases and 40 controls.

**Table 2:** Comparison of the 20 cases and 40 controls in univariate analysis with conditional logistic regression

	Cases (*n *= 20)	Controls (*n *= 40)	P
Female, n	19	34	0.27
No-low/moderate-high rheumatic disorder activity, n	11/7	20/20	0.67
TJA surgery on affected or matched joint within the last year, n	8	5	**0.03†**
- of which primary TJA, n/TJA revision, n	5/3	3/2	
- after TNFα-blockers introduction	6 of 8	5 of 5	
- after TNFα-blocker withdrawal ≥5 half-lives	1 of 6	4 of 5	0.08
Previous TJA infection, n	3	0	0.08
- of which same TJA involved, n	2	-	-
Main comorbidities, n			
- Diabetes mellitus	2	1	0.26
- Bronchiectasis	0	1	0.99
- Cirrhosis	0	0	1
- Cancer/hemopathy	0	2	0.99
- HIV	0	0	1
- Chronic renal failure	1	2	1
- Hypogammaglobulinemia	1	1	0.88
Current TNFα-blocker:			
- Infliximab/etanercept/adalimumab, n	7/5/8	13/15/12	0.70
- Duration of exposition to the current TNFα-	26.0 ± 24.1	39.0 ± 24.6	0.06
blocker (months) *			
Number of prior TNFα-blockers *	0.5 ± 0.7	0.6 ± 0.7	0.69
Total duration of exposition to any TNFα-blockers (months) *	32.0 ± 25.6	48.6 ± 25.2	0.07
Oral intake of steroids * (mg/d)	9.5 ± 7.3	5.3 ± 3.9	**0.02†**
Oral intake of steroids ≥10 mg/d, n	7	7	0.06
Intravenous infusion of steroids last year, n	2	1	0.75
Current DMARDs, n			
- Methotrexate	14	26	0.71
- Leflunomide	1	5	0.99
- Azathioprine	0	2	0.40

* mean ± standard deviation; † Results achieving a *P *value < 0.05 were considered as statistically significant.

DMARDs, disease-modifying anti-rheumatic drugs; TJA, total joint arthroplasty; TNF, tumor necrosis factor.

Regarding anti-rheumatic treatments, only increased daily steroid intake was significantly associated to TJA infection (*P *= 0.02), but no dose threshold was identified. Non-biologic disease-modifying anti-rheumatic drugs (DMARDs), in particular methotrexate (*P *= 0.71), were not significant risk factors. The distribution between TNFα blockers was similar in cases and controls (*P *= 0.70), but the duration of exposure to the current TNFα blocker and to TNFα blockers in general was longer in controls than in cases (*P *= 0.06 and 0.07, respectively).

### Risk factors of TJA infection in multivariate analysis

As shown in Table 3, two risk factors were identified in multivariate analysis: steroid intake (odds ratio (OR) = 5.0 per 5 mg/day more; 1.1 to 21.6; *P *= 0.03) and primary TJA or TJA revision for the joint subsequently infected within the past year (OR = 88.3; 1.1 to 7,071.0; *P *= 0.04).

**Table 3:** Risk factors of TJA infection using multivariate analysis with conditional logistic regression

Variable	Odds ratio	95% Confidence interval	P
- Daily steroid intake (per 5 mg/day increase)	5.0	1.1 to 21.6	0.03
- Primary TJA or TJA revision for the joint subsequently infected during the last year	88.3	1.1 to 7,071.0	0.04

TJA, total joint arthroplasty.

## Discussion

In the present study, TJA infection appears as a rare but potentially severe complication of TNFα blockers. Main risk factors are primary TJA or TJA revision for the joint subsequently infected within the past year and steroid intake.

Microbial agents identified in the present study were similar to those usually observed in TJA infections in patients having or not a rheumatic disorder [20,29,36,37] and in RA patients not exposed to TNFα blockers [38]. Nevertheless, most of the infections were related to MSSA. This was more often involved than in previous studies concerning TJA infections in patients having or not a rheumatic disorder (22 to 45%) [20,29,36,37], and in RA patients not exposed to TNFα blockers (37%) [38]. No opportunistic agent was observed, but the low number of cases does not allow conclusions about the risk of opportunistic TJA infections in this population. TNFα blockers are known to compromise local wound healing [39], so that an increased rate of polymicrobial infections could have been expected; however, the rate of polymicrobial infections (*n *= 2, 10%) was also consistent with data from the literature concerning TJA infections in patients having or not a rheumatic disorder (11 to 25%) [29,36,37], and in RA patients not exposed to TNFα blockers (15%) [38].

This study suggests that the outcome of TJA infections is particularly severe in rheumatic patients exposed to TNFα blockers, leading to hospitalization in an ICU in 20% of the cases, to death in 10% and to moderate to severe functional disability in about 40%. A high rate of bacteremia (60%) was observed in this study compared with 44% in a retrospective study assessing the natural history of TJA infections in RA patients not exposed to TNFα blockers [38]. Thus, TNFα blockers could increase the severity of the sepsis, but further controlled studies are needed to assess this hypothesis.

If TJA infections of large joints are often severe, their incidence appears rare in rheumatic patients exposed to TNFα blockers. The French national RATIO registry identified only 13 cases over two years. However, this study was not designed to estimate the incidence of TJA infections in these patients. The main objective of RATIO was to collect in an exhaustive way all over the country two rare side effects of TNFα blockers, which are opportunistic infections (including tuberculosis) and lymphomas. Severe documented bacterial infections (except pneumonias) were also collected, knowing that it was impossible to be exhaustive. Thus, we focused on specific types of severe infections such as TJA infections. Taking into account the fact that septic arthritis is frequent in RA, we were convinced that all cases of TJA infection have not been declared in the RATIO registry. But whatever it is, this study reporting 20 cases of TJA infection in patients treated with TNFα blockers is to our knowledge the largest of the literature concerning this peculiar complication.

Actually, the herein reported case-control study was designed to assess risk factors of TJA infection in patients exposed to TNFα blockers. Steroids are a classic risk factor of infection in RA patients exposed [13,16] or not [40,41] to TNFα blockers. This study confirms this data specifically for TJA infections in patients exposed to TNFα blockers. The fact that patients with TJA infection had more steroids than those without infection could also be an argument in favor of a more severe rheumatic disorder. No threshold of steroid intake was identified through this study, probably because of the small sample size.

Primary TJA or TJA revision within the past year was identified as an important risk factor of subsequent infection of this TJA. This could be due to bacterial perioperative contamination through a hematogenous way or a closed infected site, which is probably more difficult to control in the case of immunomodulation by TNFα -blockers. An infliximab-induced blood neutrophil deactivation has previously been demonstrated indeed [42]. Previous studies assessing orthopedic surgeries failed to demonstrate that the perioperative withdrawal of TNFα blockers reduced the risk of infection [24,28,43]. In the present study, TNFα blockers were less often withdrawn (stopped for more than five half-lives) before arthroplastic surgery in cases than in controls. This difference tended to reach statistical significance (*P *= 0.08), suggesting that TNFα blocker withdrawal could limit the infectious risk. No strong evidence exists but prospective controlled studies are not conceivable. In this context, withdrawal of TNFα blockers before surgery is now highly recommended by all societies of rheumatology and experts [14,44,45]. In this study, we considered five half-lives since the last administration of TNFα blockers for classifying patients as no longer exposed to the drug at the time of surgery. Recommended delays between last TNFα blocker administration and orthopedic surgery depends on each society of rheumatology. For example, the Dutch Society for Rheumatology recommends four half-lives for each TNFα blocker [45], whereas the French guidelines established in 2005 recommend two weeks for etanercept, and four weeks for infliximab and adalimumab [44]. However, it is well specified in the latter guidelines that it is a minimum and that this time has to be increased in the case of high infectious risk surgery such as TJA setup.

The present study suggests that a previous TJA infection before the introduction of TNFα blockers could be a risk factor of reoccurence, although statistical significance was not reached. In case of previous TJA infection, high reoccurence rates of TJA (10% at 3 years and 26% at 10 years) [46] have previously been reported. In RA patients exposed or not to TNFα blockers, previous surgical site infection is an identified risk factor of postoperative infection after orthopedic surgery [43] and after TJA [21]. Interestingly, the British Society of Rheumatology recommended that previous sepsis on a TJA that remains *in situ *is a definitive contraindication of TNFα blocker use [47].

Diabetes mellitus has previously been associated with an increased risk of infection after orthopedic surgery in RA [48]. The low number of cases (including only two diabetic cases) in this study did not permit to confirm this hypothesis concerning TJA infections in case of TNFα-blocker therapy. Based on data from the literature, methotrexate does not seem to modify the risk of postoperative infections after orthopedic surgery [48], and particularly after TJA [43], but previous results were heterogeneous [49,50]. Our study supports the absence of increased risk of TJA infection if methotrexate is added to anti-TNFα treatment. Regarding the type of TNFα blocker, there was no significant difference between cases and controls in our study.

The present study has several limitations and strengths. Some potential risk factors (recent skin infection, number of previous DMARDs, cumulative steroid intake, non-steroidal anti-inflammatory drugs) were not assessed because of missing data in several files. Even if it was prospective, the possible recruitment bias in the RATIO registry has already been detailed. There were only two *Staphylococcus epidermidis *infections although we would have expected more [51]. The case selection method may have led to underestimate the early less aggressive operatively-induced infections and to recruit the more severe hematogenous infections. The low number of cases has limited the statistical power of the study, and the retrospective recruitment of controls in a single center could have led to confounding factors. At the beginning of the study, no data published in the literature suggested a different risk of prosthetic infection between females and males, so we decided that controls would not be required to be of the same gender as the cases. However, a recent study highlighted a higher risk of revision due to deep infection on hip arthroplasty in males than in females [42], and gender could be a confounding factor in our study. Controls were not selected in the same way as cases (i.e., through a national study) but in one center, which may induce bias. However, the tertiary care center where controls were selected is a referral center, therefore receiving patients having the most severe rheumatic disorders, which we believe selects controls close to the cases. Furthermore, the presence of a control group including two controls per case, even if it is imperfect, increases the validity of the results. To our knowledge, this study is the first case-control study assessing risk factors of TJA infection in rheumatic patients exposed to TNFα blockers.

## Conclusions

TJA infection is a rare but severe complication in patients receiving TNFα blockers. Microbial agents do not differ from those usually identified in TJA infections, with *Staphylococcus *species involved in most cases. Two important modifiable risk factors have been identified: recent TJA setup in the previous year, that is primary TJA or TJA revision, in particular if TNFα blockers are not withdrawn before surgery, and steroid intake.

Practical implications of this study are the following. It may be preferable to perform arthroplasty, if needed, before the introduction of TNFα blockers. In cases of prosthetic surgery after the introduction of TNFα blockers, their withdrawal during the perioperative period is highly recommended. Finally, steroid intake should be reduced as low as possible in patients with both TJA and TNFα blockers.

## Abbreviations

AS: ankylosing spondylitis; CRP: C-reactive protein; DMARDs: disease-modifying anti-rheumatic drugs; IQR: interquartile range; MRSA: methicillin-resistant *Staphylococcus aureus*; MSSA: methicillin-susceptible *Staphylococcus aureus*; OR: odds ratio; PsA: psoriatic arthritis; RA: rheumatoid arthritis; RATIO: Research Axed on Tolerance of bIOtherapies; TJA: total joint arthroplasty; TNF: tumor necrosis factor.

## Competing interests

RATIO has been supported by a research grant from the INSERM (Réseau de Recherche en Santé des Populations 2003 and 2006), and by an unrestricted grant from Abbott, Shering Plough and Wyeth, but these commercial sources played no further role in the herein reported work. The authors declare that they have no competing interests.

## Authors' contributions

MG, LG and DS designed the study. MG, DG, MHG, JMB, DW, CM, and PD participated in data collection. LG and FT analyzed and interpreted the data. MG and LG drafted the manuscript. XM, MD and DS were involved in revising the manuscript critically for important intellectual content. All authors read and approved the final manuscript.
